# Characteristics and time points to inhibit ferroptosis in human osteoarthritis

**DOI:** 10.1038/s41598-023-49089-y

**Published:** 2023-12-07

**Authors:** Yangyang Xu, Zhenyu Yang, Tianming Dai, Xiang Xue, Dong Xia, Zhencheng Feng, Jian Huang, Xiaosheng Chen, Shengjie Sun, Jing Zhou, Yunmeng Dai, Jiaqi Zong, Siming Li, Qingqi Meng

**Affiliations:** 1 Jinan University, Guangzhou, Guangdong Province China; 2 Guizhou Medical University, Guiyang City, Guizhou Province China; 3 Guangzhou Institute of Traumatic Surgery, Guangzhou Red Cross Hospital of Jinan University, Guangzhou, China; 4 Xuzhou New Health Hospital, North Hospital of Xuzhou Cancer Hospital, Xuzhou City, Jiangsu Province China; 5 Department of Ultrasound Medicine, First People’s Hospital of Xuzhou City, Xuzhou City, Jiangsu Province China

**Keywords:** Osteoarthritis, Chronic inflammation, Cell death, Mitochondria

## Abstract

Ferroptosis is a form of cell death that is triggered by iron-dependent lipid peroxidation and is closely associated with osteoarthritis. The primary interventions for inhibiting ferroptosis in osteoarthritis are anti-lipid peroxidation and iron chelation. The objective of our study is to investigate the characteristics of ferroptosis in osteoarthritis and identify the optimal time points for inhibiting ferroptosis to alleviate disease progression. Ferroptosis-related alterations and markers of OA were analyzed in paired intact and damaged cartilages from OA patients by immunofluorescence, qRT-PCR, mitochondrial membrane potential and immunohistochemistry. We also compared Ferroptosis-related alterations in cartilage of mild, moderate, and severe OA (according to the modified Mankin score). In addition, we compared the effect of Fer-1 on ferroptosis and the protection of chondrocytes by detecting markers of both ferroptosis and OA by immunofluorescence, CCK8 and qRT-PCR. Ferroptosis-related alterations (GPX4 downregulation, ACSL4 upregulation, MDA, LPO accumulation, Mitochondrial membrane potential decreased) in the damaged area cartilage were more severe than those in the intact area and increased with the progression of OA. Compared with mild OA group, the activity of chondrocytes treated with Fer-1 (a ferroptosis inhibitor) was increased, mitochondrial function was improved, and ferroptosis was reduced (GPX4 upregulation, SLC7A11 upregulation, ACSL4 downregulation,), and promoted the expression of COL2A1 and inhibited the expression of MMP13. However, these changes were not observed in moderate and severe OA chondrocytes. Ferroptosis occurs in a region-specific manner and is exacerbated with the progression of human OA cartilage degeneration. Inhibition of ferroptosis might had a therapeutic effect on chondrocytes with mild OA but had no significant therapeutic effect on chondrocytes with moderate to severe OA.

## Introduction

Osteoarthritis (OA) is a degenerative disease characterized by synovial inflammation and destruction of articular cartilage^1^. The pathogenesis of OA is complex, involving different types of cell death, including ferroptosis^2^. Recent studies have confirmed that ferroptosis promotes OA progression^3^. In 2020, Yao demonstrated for the first time that chondrocytes underwent ferroptosis under inflammatory and iron overload conditions, and the use of ferroptosis inducers resulted in increased matrix metalloproteinase 13 (MMP13) expression and reduced type II collagen(COL2A1) expression in chondrocytes in a mouse OA model^4^. MMP13 is a member of the matrix metalloproteinases(MMPs), which are involved in the breakdown of extracellular matrix in OA, and COL2A1 is the basis for articular cartilages^5^. Hence, decreased COL2A1 and increased MMP13 secretion by chondrocytes are major biomarkers and contributors of OA.

Ferroptosis, first reported by Dixon et al. in 2012, is a form of non-apoptotic cell death driven by iron-dependent lipid reactive oxygen species (ROS) and accelerated by the accumulation of lipid peroxides, ultimately leading to oxidative damage to phospholipid membranes and cell death^6^. Ferroptosis is characterized by the inactivation of the antioxidant glutathione peroxidase 4(GPX4) and the accumulation of lipid ROS^7^. In 2022, Miao found a significant decrease in enzyme GPX4, a key indicator of ferroptosis in human OA^8^. Acyl-CoA synthetase long-chain family member 4(ACSL4) also is a biomarker and contributor of ferroptosis^9^. The inhibition of Solute Carrier Family 7 Member 11(SLC7A11), a subunit of cystine/glutamate antiporter, resulted in the depletion of intracellular glutathione, iron-dependent lipid peroxidation, and subsequent ferroptosis^10^. As a tumor suppressor, P53 is not only a key regulatory element in apoptosis but also a critical molecule in ferroptosis as it inhibits the production of SLC7A11^11^. Indeed, much evidence indicates that the Fenton reaction is the main source of ROS in the occurrence of ferroptosis, and the mitochondrion is a major organelle for cellular ROS production^12^. Wang et al.^13^ found that the development of ferroptosis is closely linked to mitochondrial dysfunction, and the main mechanism may involve irreversible damage to mitochondrial morphology and metabolic function caused by iron overload, ROS, and lipid peroxide (LPO) accumulation, thus promoting the progression of ferroptosis. Malondialdehyde (MDA) is a product of lipid peroxidation and represents the level of lipid peroxidation^14^. Mitochondrial membrane potential (MMP) decrease conforms to the representative features of the mitochondria in ferroptosis^15^. However, the characteristics of ferroptosis in chondrocytes remain unclear.

In present study, our study strongly suggests that ferroptosis in patients with OA, which was present in a region-specific manner in different cartilage damaged areas in one same joint. Some researchers have certificated that the gene expression profile of chondrocytes in the same OA articular cartilage varies in a region-specific manner^16^. However, the relationship between chondrocyte ferroptosis and the degree of cartilage histopathological alterations in human OA are unclear. OA has numerous grading criteria, for example, knee OA includes the Kellgren–Lawrence (K–L) grading, Recht grading, and Mankin score, among others^17–19^. The K–L grading score is a grading scale for X-ray, the Recht grading score is a grading scale for MRI, and the Mankin score is a grading scale for histopathological changes in the cartilage. In this study, we classified human OA samples into three grades, mild, moderate, and severe, based on the Mankin score, with respect to the degree of local cartilage damage from the same joint. The ferroptosis-related alterations and markers of OA in the regions of intact and damaged cartilage in the same joint. We also compared markers of Ferroptosis and OA in cartilage of mild, moderate, and severe OA. The therapeutic effects of Ferrostatin-1 (Fer-1) on chondrocytes derived from mild, moderate, and severe OA were observed.

Some findings suggest that the inhibition of ferroptosis may have a therapeutic effect on the progression of OA^20^. Indeed, Wang et al.^21^ found that Fer-1 alleviates the IL-1β-induced inflammatory response and the degradation of the extracellular matrix in rat chondrocytes and has a protective effect on MMP. However, these results have not been previously demonstrated in human OA chondrocytes. Furthermore whether inhibition of ferroptosis has consistent effects on chondrocytes with different degrees of injury has not been addressed so far. However, this question is very important for guiding the treatment of patients with different OA stages in clinic. In this study, we investigated the manifestation of ferroptosis in chondrocytes with different degrees of injury in osteoarthritis and elucidated the therapeutic effects of ferroptosis inhibitors on chondrocytes at different time points during the progression of OA.

## Materials and methods

### Ethics approval

The study was conducted according to the guidelines of the Declaration of Helsinki, and approved by the Ethical Committee of Guangzhou Red Cross Hospital of Jinan University (2021-187-01). Informed consent was obtained from all subjects involved in the study.

### Cartilage samples

Human cartilage tissues with OA were obtained from nine patients undergoing total knee replacement (TKR) surgery. Normal cartilage tissue was obtained from one patient undergoing arthroscopy. Knee OA was diagnosed based on clinical and radiological evaluation. All nine cartilage samples collected from patients with OA were divided into three groups according to a modification of the Mankin score (grade I = mild OA scored 0–5, grade II = moderate OA scored 6–10, and grade III = severe OA scored 11–14). Cartilage specimens were taken from the same joint, with the medial tibial plateau as the damaged area and the lateral tibial plateau as the intact area (n = 3). The clinical characteristics of the 10 study patients are shown in Supplementary Table S1.

### Isolation and culture of human chondrocytes

Human primary chondrocytes were isolated from articular cartilage fragments, which were dissected from the femoral condyles and tibial plateau of patients. Briefly, cartilage fragments were first digested with 0.25% trypsin (Gibco, CA, USA) at 37 °C for 30 min, before fully digesting using 0.2% collagenase II (Sigma, St. Louis, MO, USA) at 37 °C for 6 h. After dispensing the digested cartilage fragments through a 70-μm cell strainer, primary chondrocytes were cultured with Dulbecco’s modified Eagle’s medium-F12 (DMEM/F12; Gibco, Hangzhou, China) supplemented with 10% fetal bovine serum (Gibco, CA, USA) and 1% penicillin/streptomycin cocktail (Gibco, CA, USA) under standard conditions (37 °C, 5% CO2) for 5–10 days. Human primary chondrocytes were used for lipid peroxidation and MDA detection. Primary chondrocytes were used in this experiment.

### Immunofluorescence staining

Human chondrocytes were seeded on a 24-well plate and cultured overnight. The cells were treated with Fer-1, then fixed in 4% paraformaldehyde (Beyotime, Shanghai, China), permeabilized with 0.2% Triton-X (Beyotime, Shanghai, China), and blocked with 2% bovine serum albumin (Sigma, St. Louis, MO, USA) for 0.5 h at room temperature. After overnight incubation with GPX4 (1:50; Zen-bio, R24461) and ACSL4 (1:20; Proteintech, 22401-1-AP) at 4 °C, the cells were incubated with a Cy3-conjugated secondary antibody (1:50;Servicebio, Wuhan, China) and FITC-conjugated secondary antibody (1:20;Servicebio, Wuhan, China) at room temperature for 1 h, followed by Hoechst 33258 (10 μg/mL) for 5 min. Images were acquired using a fluorescence microscope (Nikon, Eclipse-Ti): fluorescence excitation/emission 550/352 nm for GPX4, 458/358 nm for ACSL4, and 352⁄461 nm for Hoechst.

### Cell viability assay

Cell viability was measured by Cell Counting Kit-8 (Beyotime, Shanghai, China). Further details are described in Supplementary Materials and methods. Briefly, human chondrocytes were seeded onto 96-well plates (5,000 cells per well). The next day, the cells were treated with the ferroptosis inhibitor Fer-1 (MCE, NJ, USA). Subsequently, the cells were exposed to 100 μl of a 10% CCK-8 solution for 1 h at 37 °C, 5% CO_2_ in an incubator. The absorbance at 450 nm was measured using a microplate reader (GloMax Multi Plus, Promega).

### Lipid peroxidation assay

For the lipid peroxidation assay, human primary chondrocytes were stained with 10 μM BODIPY 581/591 C11 (Invitrogen, D3861) at 37 °C for 30 min. After incubation, the cells were washed with phosphate-buffered saline (PBS), harvested by trypsinization, and resuspended in 200 μl PBS. Lipid peroxidation was assessed using the flow cytometer BD FACSVerse (Becton Dickinson) with the FITC filter. A minimum of 10,000 cells were analyzed per sample. Data analysis was performed using FlowJo v10 (BD Bioscience).

### Histological and immunohistochemical staining

Cartilage tissue samples were fixed in 4% buffered paraformaldehyde and subsequently decalcified with buffered EDTA (20% EDTA, pH 7.4). The tissues were embedded in paraffin, sectioned, and stained with safranin O/fast green.

For immunohistochemistry staining, the sections were deparaffinized, antigen retrieved, and blocked. After incubation with primary antibodies against COL2A1 (1:100; Servicebio, GB11021), MMP13 (1:100; Servicebio, GB11247), GPX4 (1:50; Zen-bio, R24461), and ACSL4 (1:20; Proteintech, 22401-1-AP), the sections were incubated with secondary antibody (Servicebio, G1215), followed by counterstaining with hematoxylin and visualized by DAB (Servicebio, G1212). The images were captured by a microscope (Nikon, Eclipse Ci), and the number of positive cells/total cells per field in each group was counted under 200× magnification.

### Malondialdehyde assay

Human primary chondrocytes were lysed, and the MDA concentration was measured by a Lipid Peroxidation MDA Assay Kit (Beyotime, S0131S) according to the manufacturer’s instructions at an absorbance of 560 nm. The protein concentrations were quantified using a BCA Protein Assay Kit (Beyotime, P0012S) to normalize the MDA content.

### MMP detection

The MMP of chondrocytes was measured by fluorescence microscopy using a Mitochondrial Membrane Potential Assay Kit with JC-1 staining (Beyotime, Shanghai, China) according to the manufacturer’s protocol. Briefly, human chondrocytes were seeded in a six-well plate with the indicated treatment. Subsequently, the cells were incubated with JC-1 staining solution for 20 min at 37 °C and imaged with a fluorescence microscope (Nikon, Eclipse-Ti) on fluorescence excitation/emission maxima: 514/529 nm, monomer form; 585/590 nm J-aggregate form.

### RNA extraction and quantitative real-time polymerase chain reaction (qRT-PCR) analysis

RNA was extracted using TRIzol (Invitrogen), and total RNA was reverse transcribed into cDNA using a reverse transcription kit (Takara, Beijing, China). Real-time quantitative polymerase chain reactions were performed with the SYBR Green RT-PCR reagent (Takara). Gene expression levels were normalized to GAPDH using the 2^−△△Ct^ method. The primers are listed in Table S2.

### Statistical analysis

Bars and error bars are presented as the mean ± standard deviation (SD) of at least three independent replicates. Statistical analyses were performed using GraphPad Prism 9.0.0 (GraphPad Software). For the comparation of mean values between two groups, paired or unpaired two-tailed Student’s t test was used. One-way or two-way analysis of variance (ANOVA) followed by Tukey’s post-hoc tests were used to assess the statistical significance of the mean values of more than two groups. Data were presented as mean values ± SD. The statistical significance was set at P < 0.05.

## Results

### Ferroptosis occurs in human OA in a region-specific manner.

**Figure 1:**
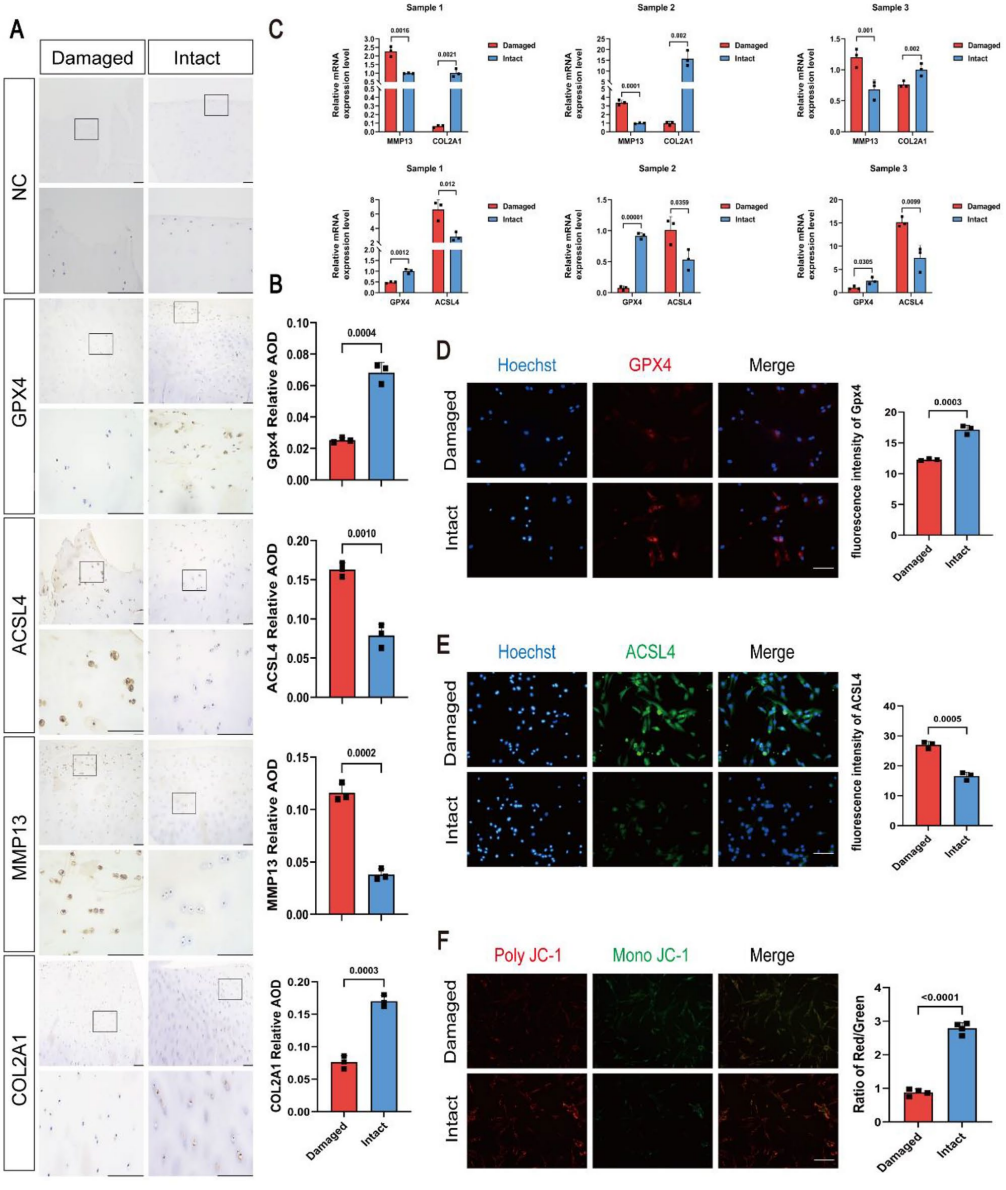
Ferroptosis occurs in OA cartilage in a region-specific manner. (**A**) Representative immunohistochemical (IHC) staining of NC (Negative Control), GPX4, ACSL4, MMP13, and COL2A1 in paired intact and damaged human OA cartilages. Scale bars: 100 μm (top) and 20 μm (bottom). (**B**) Quantification of immunohistochemical analysis (n = 3). (**C**) RT-qPCR was used to detect the mRNA expression of MMP13, COL2A1, GPX4, ACSL4 in human OA cartilage (n = 3). (**D**) Representative immunofluorescence staining and quantification analysis of GPX4 in human OA cartilage (n = 3). Scale bar: 50 μm. (**E**) Representative immunofluorescence analysis of ACSL4 in human OA cartilage (n = 3). Scale bar: 50 μm. (**F**) JC-1 staining was performed to monitor mitochondrial membrane potential (Δψm). The ratio of the red/green fluorescence indicated the degrees of mitochondrial damage (n = 3). Scale bar: 50 μm. Data are expressed as means + SD. For all experiments p-values calculated by unpaired two-tailed Student’s t test, p < 0.05, a statistically significant difference.

To determine the correlation between ferroptosis-related alterations and OA, we collected three sets of cartilage from patients with OA and examined the expression of Markers of OA (MMP13, COL2A1) and ferroptosis (GPX4, ACSL4) in human cartilage. Immunohistochemistry results showed that the expression of COL2A1 and GPX4 were significantly higher (2.23 folds, 2.72 folds) in the intact region than in the damaged region of OA cartilage, while the expression of MMP13 and ACSL4 were higher (3.05 folds, 2.06 folds) in the damaged region than in the intact region (Fig. 1A, B). This finding suggests that chondrocyte damage is related to the increasing degeneration of cartilage, and a clear difference in ferroptosis-related indicators in damaged regions of human OA cartilage compared to those in intact regions.

To further explore the question of ferroptosis-related alterations and OA, we measured the expression of markers of OA and ferroptosis by Quantitative reverse-transcription PCR (qRT-PCR) (COL2A1, MMP13, GPX4 and ACSL4) and Immunofluorescence staining (GPX4 and ACSL4).Consistent with the observations, qRT-PCR results showed lower COL2A1, GPX4 and higher MMP13, ACSL4 in the damaged region than in the intact region (Fig. 1C). Immunofluorescence staining results showed that the GPX4 expression decreased 0.7 fold in damaged area chondrocytes than intact area, while the results of ACSL4 were opposite (Fig. 1D, E).

Because the dysfunction of mitochondria is often observed when ferroptosis occurs, the mitochondrial membrane potential (Δψm) in chondrocytes was detected^15,22^.

The Δψm maintains an electrochemical gradient across the mitochondrial membrane, which collapses in the process of cell death. The changes of Δψm were detected using the Mitochondrial membrane potential assay kit with JC-1 staining. The unique cationic dye, JC-1 (5, 5,6, 6-tetrachloro-1, 1,3, 3 tetraethylbenzimidazolylcarbocyanine iodide) is a membrane potential-sensitive probe that forms aggregates in the mitochondria with higher membrane potential showing fluorescent red at 590 nm. With collapsed Δψm, monomers of the JC-1 remain in the cytosol of cells and emit a fluorescent green at 530 nm. The intensity of the fluorescence is proportionally reflecting the Δψm level. The ratio between 530 and 590 nm emission manifest the fettle of Δψm^23^. The red/green fluorescence ratio indicates the level of Δψm. Compared to chondrocytes of intact area, the intensity of red fluorescence (PolyJC-1) of chondrocytes of damaged area was significantly weaker, while the intensity of green fluorescence (mono JC-1) was significantly stronger (Fig. 1F). The ratio of red to green fluorescence intensity in damaged area was significantly decreased than that of intact area, indicating an increase in the degree of mitochondrial damage. Therefore, our study suggested that ferroptosis occurs in a region-specific manner in human OA.

### Ferroptosis increased with increasing cartilage degeneration in human OA

**Figure 2:**
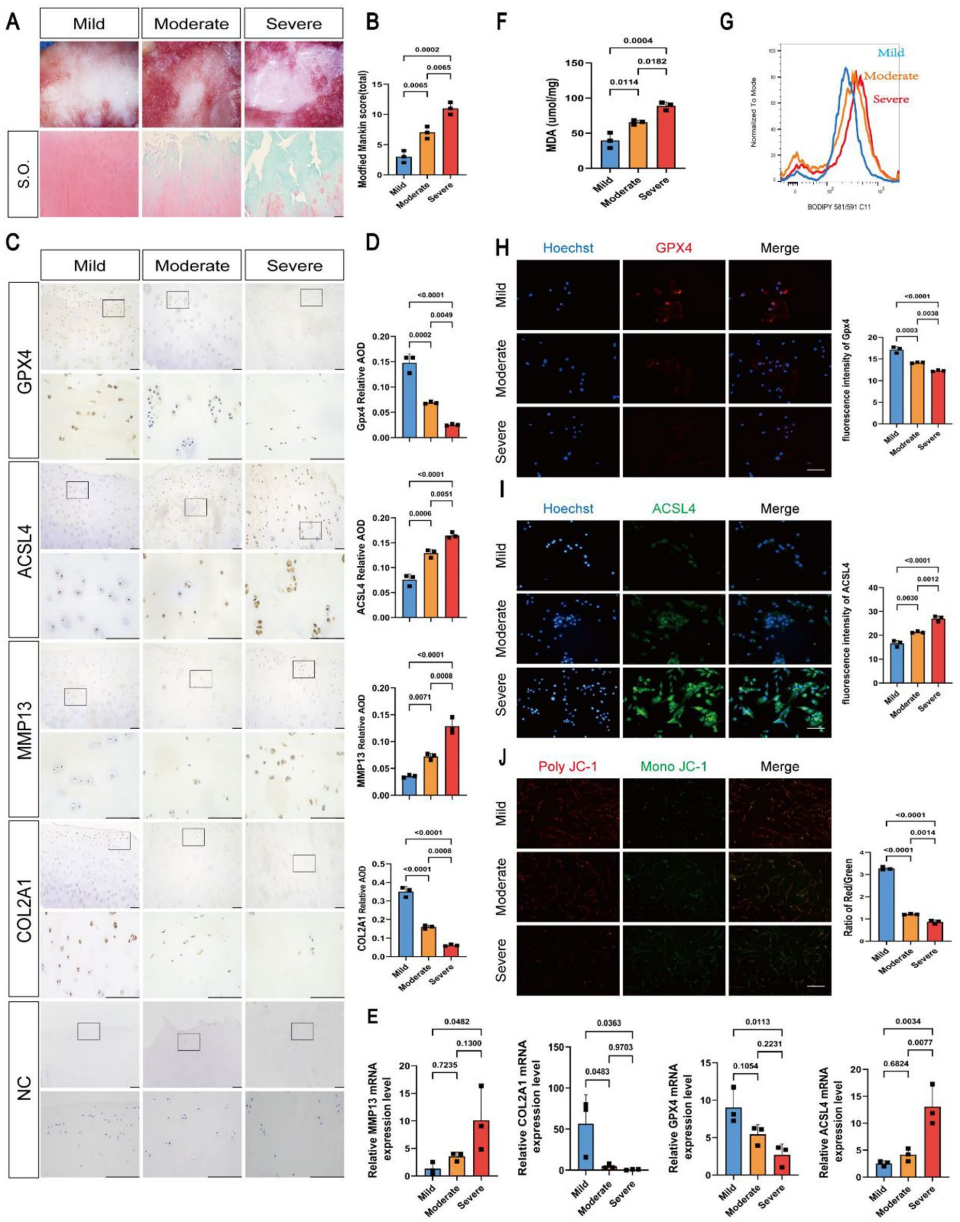
Ferroptosis increased with increasing cartilage degeneration in human OA. (**A**) Safranin O-fast green (S.O) staining of OA cartilages. (**B**) Modified Mankin score of OA cartilage. (**C**) Representative IHC staining of NC (Negative Control), GPX4, ACSL4, MMP13, and COL2A1 in human OA cartilages. Scale bars: 100 μm (top) and 20 μm (bottom). (**D**) Quantification of IHC analysis (n = 3). (**E**) RT-qPCR was used to detect the mRNA expression of MMP13, COL2A1, GPX4, ACSL4 in human OA cartilage. (n = 3). (**F**) MDA contents of human OA cartilage (n = 3). (**G**) Flow cytometry analysis of lipid peroxide level of human OA cartilage. (**H**) Representative immunofluorescence staining and quantification analysis of GPX4 in human OA cartilage (n = 3). Scale bar: 50 μm. (**I**) Representative immunofluorescence staining and quantification analysis of ACSL4 in human OA cartilage (n = 3). Scale bar: 50 μm. (**J**) Mitochondrial membrane potential was detected by JC-1 assay (n = 3). Scale bar: 50 μm. Data are expressed as means + SD. For all experiments p-values calculated by ANOVA analysis, p < 0.05, a statistically significant difference.

To investigate ferroptosis-related alterations and its relationship to the extent of degradation of cartilage in articular cartilage. All cartilage samples were divided into three groups according to the modified Mankin scale: grade I were samples with mild OA (scored 0–5), grade II were samples with moderate OA (scored 6–10), and grade III were samples with severe OA (scored 11–14), where the higher the score, the more severe the cartilage degeneration. According to safranin O/fast green staining, the joint surface in the mild OA was relatively smooth, with clearly visible cartilage structures and abundant proteoglycan. The articular cartilage in the moderate OA was significantly degraded with the loss of proteoglycan. The cartilage destruction in the severe OA was the most obvious, and the proteoglycan content was the lowest in the severe group. Therefore, the modified Mankin scores of human cartilage in the severe OA were significantly higher than those in the other two groups (Fig. 2A, B).

First, we collected nine cartilages from patients with OA, and examined the expression of MMP13, COL2A1, GPX4 and ACSL4 in human cartilage. In mild OA cartilage samples, the expression of COL2A1 (mild/moderate 2.18 folds, mild/severe 5.74 folds) and GPX4 (mild/moderate 2.14 folds, mild/severe 5.92 folds) was significantly higher than in the other groups, in addition, the expression of MMP13 (mild/moderate 0.5 fold, mild/severe 0.28 fold) and ACSL4 (mild/moderate 0.59 fold, mild/severe 0.46 fold) was significantly lower than that in the other groups. In moderate and severe OA cartilage samples, the expression of GPX4 and COL2A1 decreased, while the expression of MMP13 and ACSL4 increased (Fig. 2C, D). These data indicate that GPX4 is intensely expressed in cartilage with a low Mankin scale score, and GPX4 expression decreases in parallel with increasing MMP13 expression, whereas ACSL4 is the opposite.

To further analyze relationship between ferroptosis-related alterations and the extent of degradation of cartilage. We measured the expression of markers of OA and ferroptosis by qRT-PCR (COL2A1, MMP13, GPX4 and ACSL4) and Immunofluorescence staining (GPX4 and ACSL4). qRT-PCR results shown that the expression of GPX4, COL2A1 decreased with increasing expression of MMP13, ACSL4 (Fig. 2E). Immunofluorescence staining results showed that the levels of GPX4 decreased with increasing cartilage degradation, while the levels of ACSL4 increased with increasing cartilage degradation (Fig. 2H, I). Measurements of MDA using commercial kits showed that MDA levels increased with increasing cartilage degradation (mild:moderate:severe = 1:1.6:2.2) (Fig. 2F). The levels of LPO, another indicator of ferroptosis detected by flow cytometry, showed the same trend (Fig. 2G). We also examined the MMP, and the results showed that the ratio of red to green fluorescence was highest in mild OA and lowest in the severe group (Fig. 2J), indicating mitochondrial damage increased with increasing cartilage degradation. These data suggest that ferroptosis increased with increasing cartilage degeneration in human OA.

### Inhibition of ferroptosis can improve chondrocyte function in mild OA, but not moderate and severe OA

**Figure 3:**
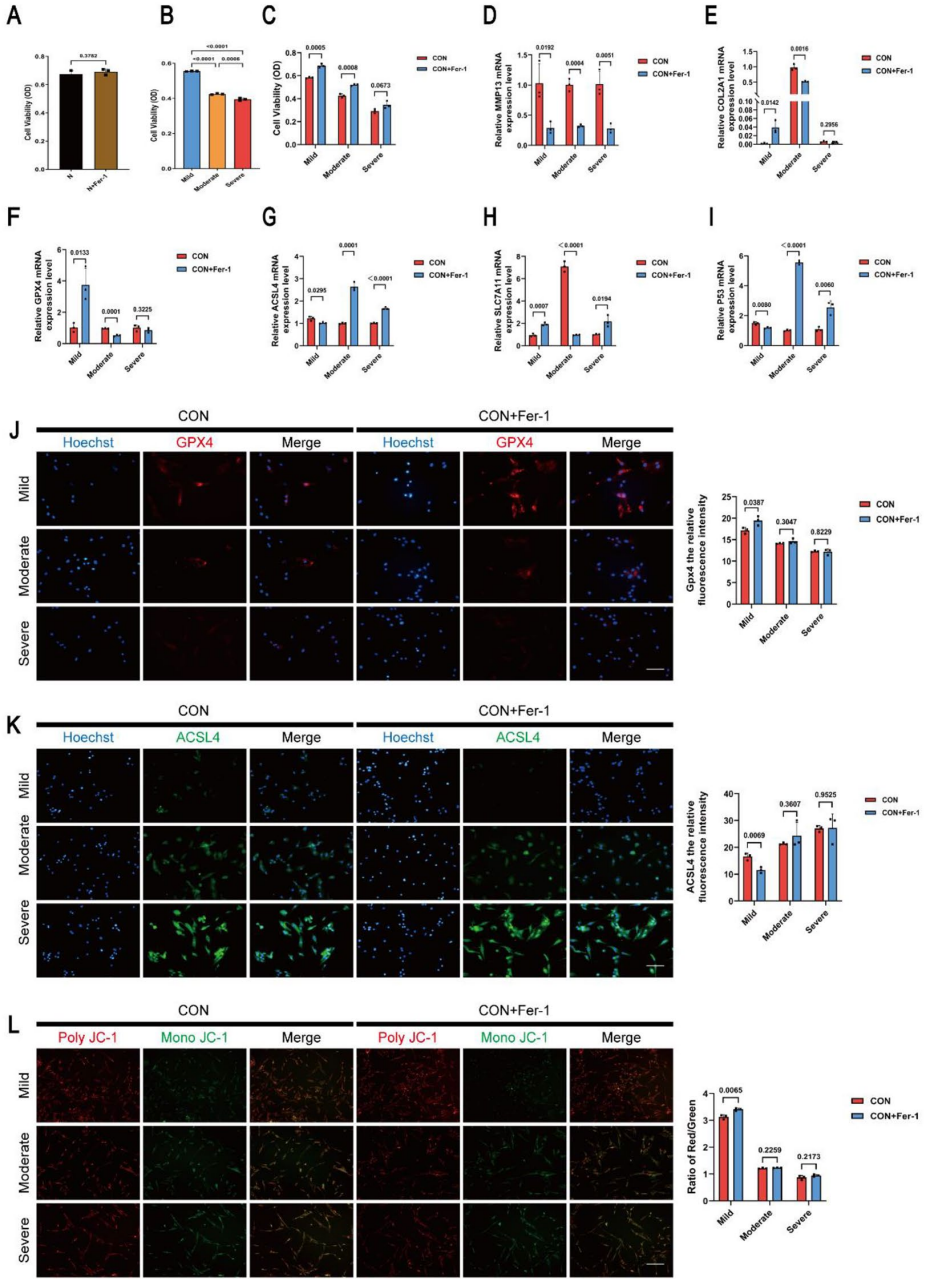
Inhibition of ferroptosis can improve chondrocyte function in mild OA, but not moderate and severe OA. (**A**) The effects of Fer-1 (2 μM) on the viability of human normal chondrocytes after 24 h of treatment was determined by CCK-8 assay. (**B**) Human chondrocyte viability was determined by CCK-8 assay. (**C**) The viability of chondrocytes was determined by CCK-8 assay following treatment of chondrocytes of each group with Fer-1 (2 μM) for 24 h. (**D**–**I**) The mRNA expression levels of MMP13, COL2A1, GPX4, ACSL4, SLC7A11 and P53 were detected by RT-qPCR following treatment of chondrocytes of each group with Fer-1 (2 μM) for 24 h. (**J**) Representative immunofluorescence staining and quantification analysis of GPX4 in the chondrocytes of each group treated with Fer-1 (2 μM) for 24 h. (**K**) Representative immunofluorescence staining and quantification analysis of ACSL4 in the chondrocytes of each group treated with Fer-1 (2 μM) for 24 h. (**L**) Mitochondrial membrane potential was detected by JC-1 assay following treatment of chondrocytes of each group with Fer-1 (2 μM) for 24 h. Scale bar: 50 μm. Data are expressed as means + SD. For all experiments p-values calculated by unpaired two-tailed Student’s t test, p < 0.05, a statistically significant difference.

To further investigate the effects of inhibition of ferroptosis on OA progression, we determined the optimal dosages of Fer-1 (2 μM and 5 μM) based on literature reports^24,25^ and CCK8 assays, and the ferroptosis-related alterations and markers of OA were detected following treatment of chondrocytes of each group with Fer-1 for 24 h. The viability of chondrocytes was determined by CCK-8 assay. CCK-8 results showed that Fer-1 had no significant effects on normal human chondrocytes viability (Fig. 3A). The activity of chondrocytes derived from the mild OA in vitro was significantly higher than that of chondrocytes derived from the other two groups (Fig. 3B). Furthermore, the inhibition of ferroptosis increased chondrocytes viability in the mild and moderate OA but not in the severe OA (Fig. 3C). These results suggest that the therapeutic effect of fer-1 on chondrocytes viability is not universal and may only have a therapeutic effect on chondrocytes during mild and moderate OA.

The decreased COL2A1 and increased MMP13 secretion by chondrocytes are major biomarkers of and contributors to OA^26,27^. As can be seen in (Fig. 3D, E) Fer-1 inhibited the expression of MMP13, while augmenting the expression of COL2A1 and exerting a rescuing effect in the mild OA. However, this phenomenon of rescuing effect was not observed in the moderate and severe OA.

SLC7A11 and GPX4 were regarded as being the well-accepted biomarkers of ferroptosis, which are the relative downstream regulators of ferroptosis. P53 is one of the multitudinous upstream regulatory genes that acts on SLC7A11^28^. P53/SLC7A11/GPX4 has been proven to be a classic signaling pathway of ferroptosis^29^. We measured the ferroptosis-related biomarkers at the RNA and protein level. qRT-PCR suggested that Fer-1 reversed the increased expression of ACSL4 and P53 (Fig. 3G, I) and the decreased expression of GPX4 and SLC7A11 (Fig. 3F, H) in the mild OA, while there were no significant differences before and after Fer-1 treatment in the moderate and severe OA. In addition, immunofluorescence staining showed that fer-1 enhanced the intensity of red fluorescence (GPX4) and attenuated the intensity of green fluorescence (ACSL4) in mild OA (Fig. 3J, K), this phenomenon of rescuing effect was not observed in the moderate and severe OA. All the phenomena were representative events in the ferroptosis progression. Collectively, this data proves that Fer-1 might mitigate chondrocytes damage in mild OA by inhibiting ferroptosis via the P53/SLC7A11/GPX4 signaling pathway. The decrease in MMP was partially restored and rescued the mitochondrial dysfunction to a certain extent after Fer-1 treatment in the mild OA, but no restorative effects were observed in the moderate and severe OA (Fig. 3L). In addition, we observed that Fer-1 (5 μM) had similar effects on chondrocytes in the mild, moderate, and severe OA as Fer-1 (2 μM) in vitro (Supplementary Fig. S1). To summarize, these data suggest that the inhibition of ferroptosis may be effective for treating mild OA but has no significant effects on chondrocytes with moderate to severe OA.

## Discussion

Previous studies have suggested that OA is a multifactorial disease, and all pathogenic factors would lead to similar cartilage degeneration patterns; that is, in the same joint, chondrocytes survive in the same intra-synovial environment, and the change in cartilage in the joint should be consistent^30^. Recent studies have confirmed that ferroptosis promotes OA progression^31^. However, the characteristics of ferroptosis in OA remain unclear. In the present study, we compared intact and damaged regions of OA cartilage from the same joint and confirmed mild manifestations of ferroptosis and OA in the intact area and aggravation in the damaged area both in vivo and in vitro. The results suggest that the ferroptosis-related indicators in OA cartilage change in a region-specific manner. Tomoo Sato et al. found that the gene expression profiles of chondrocytes in OA cartilage change in a region-specific manner, including MMP13 and COL2A1^16^. Iron overload induces OA-like changes in chondrocytes by triggering inflammation, oxidative stress, and disruption of the extracellular matrix, thereby promoting the progression of OA^20^. Excessive iron disrupts cellular iron metabolism and impairs chondrocyte function, resulting in the accumulation of iron and ROS within the cells. This leads to oxidative stress and a decrease in the production of COL2A1, which can ultimately lead to a form of programmed cell death known as ferroptosis^32^. Oxidative stress, caused by the accumulation of ROS, can activate the mitochondrial apoptotic pathway, thereby promoting the expression of MMPs, which are enzymes responsible for degrading cartilage^33^. Additionally, it has been suggested that the accumulation of ROS during ferroptosis can independently induce the expression of MMP13 and further decrease the expression of COL2A1^4^. Iron overload also triggers the expression of proinflammatory factors, which in turn stimulate the synthesis of MMPs, particularly MMP13^34–36^. MMP13 plays a crucial role in cartilage degradation by specifically cleaving COL2A1^37^. COL2A1, a major component of the cartilage matrix, is downregulated as a result of matrix degradation^38^. Therefore, it may be inappropriate to select the same approach for different areas of injury in OA treatment, and instead, the approach should be selected based on different degrees of cartilage damage.

To choose a more precise method for treating OA, we investigated the differences in ferroptosis-related alteration between different grades of OA. We evaluated the histology of OA cartilage sections and classified them as mild, moderate, and severe according to the modified Mankin score. We found that the extent of ferroptosis in chondrocytes was consistent with the severity of OA, which was consistent with the results of the in vitro experiments. GPX4 and ACSL4 have been identified as crucial factors in the initiation of ferroptosis in OA, and their involvement is closely linked to the progression of the disease. GPX4 serves as a protective factor against lipid peroxidation, while ACSL4 plays a significant role in regulating lipid metabolism. These two components are central to the observed changes in OA. It is important to note that our study primarily focused on ferroptosis, but we acknowledge the multifaceted nature of OA, which includes alterations in metabolism during advanced stages and the impact of different forms of cell death on GPX4 and ACSL4^39^. GPX4 has been demonstrated to act as a negative regulator in various modes of cell death, such as apoptosis, necrotic apoptosis, and pyroptosis^40–42^. Additionally, ACSL4 has been implicated in autophagy and can also contribute to the apoptotic process by neutralizing non-esterified fatty acids^43,44^. Although these key factors are involved in multiple forms of cell death and degeneration, our study is the first to demonstrate the alteration of GPX4, a crucial regulator of ferroptosis, and ACSL4, a sensitive biomarker, in response to increased cartilage degradation at different stages of OA.

Ferrostatin-1 is an efficient ferroptosis-specific inhibitor, which functions by eliminating the initiating alkoxyl radicals and other rearrangement products produced by ferrous iron from lipid hydroperoxides^45^. In our study, Fer-1 inhibited the expression of MMP13, while augmenting the expression of COL2A1, and maintained the dissipation of the MMP in chondrocytes derived from mild OA, However, no significant effects were observed on chondrocytes derived from moderate and severe OA. The mechanism of this phenomenon may be that in chondrocytes with mild OA, mitochondrial damage is reduced, cellular antioxidant function is easily restored, and membrane potential is easily maintained, however, in chondrocytes with moderate to severe OA, mitochondrial damage is irreversible. In our study, Fer-1 maintained the dissipation of MMP in mild OA chondrocyte ferroptosis and partially rescued mitochondrial dysfunction, however, this phenomenon was not observed in moderate and severe OA, which also confirmed the above hypothesis. Studies have shown that in a pathological state, the antioxidant defense system cannot remove excessive ROS, which results in oxidative stress and even apoptosis^46^. The overproduction of ROS leads to mitochondrial dysfunction, which, together with mitochondrial DNA damage, contributes to cartilage degeneration^47^. This is consistent with our observation of excessive ROS accumulation in the chondrocytes with severe OA. The abnormal alteration of MMP is a symbolization of mitochondrial dysfunction, which has recently been acknowledged as a marker of ferroptosis^48,49^. Mitochondrial dysfunction upregulates matrix metalloproteinase levels, leading to matrix degradation, and promotes the production of oxidative stress and inflammatory mediators. Ultimately, these pathological changes form a vicious cycle that further causes chondrocyte apoptosis and inflammation, thereby promoting the occurrence of OA^50,51^. In conclusion, the pathogenesis of OA is complex and involves many factors. Studies have shown that various modes of cell death occur in early OA. However, complex pathogenesis may be found in moderate and severe OA, including inflammation, oxidative stress, irreversible damage to chondrocytes, and degradation of extracellular matrix, which further lead to cartilage degeneration^52^.

In summary, we demonstrated the occurrence of ferroptosis in OA cartilage in a region-specific manner. Furthermore, it was found that the ferroptosis increased with increasing cartilage degeneration in OA. To the best of our knowledge, for the first time, the inhibition of ferroptosis might had a therapeutic effect on chondrocytes with mild OA but had no significant therapeutic effect on chondrocytes with moderate to severe OA.

### Supplementary Information

Supplementary Information.

## Data Availability

The data that support the findings of this study are available from the corresponding author upon request.

## Abbreviations

OA: Osteoarthritis
GPX4: Glutathione peroxidase 4
ACSL4: Acyl-CoA synthetase long-chain family member 4
SLC7A11: Recombinant Solute Carrier Family 7, member 11
P53: Cellular tumor antigen p53
MMP13: Matrix metalloproteinase 13
COL2A1: Type II collagen
MDA: Malondialdehyde
LPO: Lipid peroxidation
Fer-1: Ferrostatin-1
