# Supplementary material for: Characteristics and time points to inhibit ferroptosis in human osteoarthritis
Source: Sci Rep. 2023 Dec 7;13:21592. doi: 10.1038/s41598-023-49089-y (PMC10703773; doi:10.1038/s41598-023-49089-y)
Supplement: Supplementary file 1 — Supplementary Information. [file 41598_2023_49089_MOESM1_ESM.docx]

**1. Supplementary Figure**

**1.1 Supplementary Figure S1**


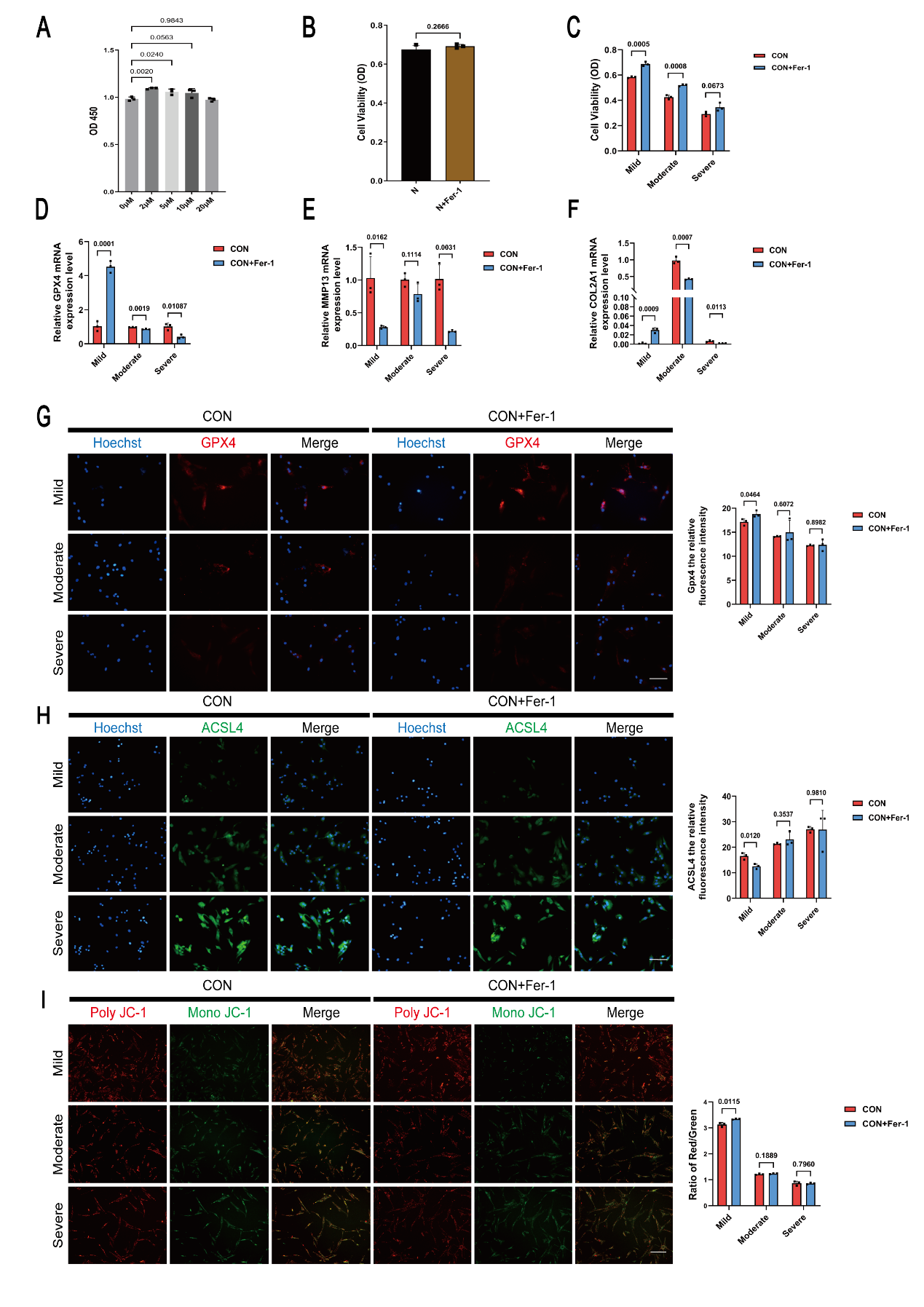


**Supplementary** **Figure S1. Inhibition of ferroptosis by Fer-1(5μM) can improve chondrocyte function in mild, but not moderate and severe human OA.** **(A) The effect of different concentrations of Fer-1 on OA chondrocyte viability was determined by CCK-8 assay.（B）**The effects of Fer-1 (5μM) on the viability of human normal chondrocytes after 24h of treatment was determined by CCK-8 assay. **(C)** The viability of chondrocytes was determined by CCK-8 assay following treatment of chondrocytes of each group with Fer-1(5μM) for 24h. **(D-F)** The mRNA expression levels of MMP13, COL2A1, GPX4 were detected by RT-qPCR following treatment of chondrocytes of each group with Fer-1(5μM) for 24h. **(G)** Representative immunofluorescence staining and quantification analysis of GPX4 in the chondrocytes of each group treated with Fer-1 (5μM) for 24h.**(H)** Representative immunofluorescence staining and quantification analysis of ACSL4 in the chondrocytes of each group treated with Fer-1 (5μM) for 24h.**(I)** Mitochondrial membrane potential was detected by JC-1 assay following treatment of chondrocytes of each group with Fer-1(5μM) for 24 h . Scale bar: 50μm. Data are expressed as means + SD.

**2 Supplementary Table**

**2.1 Supplementary Table S1. General characteristics of the cartilage donors.**

| **No.** | **Gender/Age Joint** | **Cartilage source K/L (grade)** |
| --- | --- | --- |

1 M/66 knee Lateral femoral condyle II

2 F/67 knee Lateral femoral condyle II

3 F/68 knee Lateral femoral condyle II

4 M/66 knee Medial femoral condyle III

5 F/67 knee Medial femoral condyle IV

6 F/58 knee Medial femoral condyle IV

7 F/63 knee Medial tibial plateau IV

8 F/64 knee Medial tibial plateau IV

9 F/71 knee Medial tibial plateau IV

10 M/28 knee Femoral condyles I

K/L = Kellgren/Lawrence; NO.10: Normal cartilage

**2.2 Supplementary Table S2.** **RT-qPCR Primers used in this study.**

| Name | Origin | Forward (5’→3’) | Reverse (5’→3’) |
| --- | --- | --- | --- |

GPX4 human GAGGCAAGACCGAAGTAAACTAC CCGAACTGGTTACACGGGAA

ACSL4 human CATCCCTGGAGCAGATACTCT TCACTTAGGATTTCCCTGGTCC

SLC7A11 human TCTCCAAAGGAGGTTACCTGC AGACTCCCCTCAGTAAAGTGAC

P53 human CCCAAGCAATGGGATGATTTGA GGCATTCTGGGAGCTTCATCT

MMP13 human TTGAGCTGGACTCATTGTCG CGCGAGATTTGTAGGATGGT

COL2A1 human GGACGATCAGGCGAAACC CCAGCAAAGGCGGACATG
